# Le déficit immunitaire commun variable à révélation tardive par des manifestations digestives: à propos d’un cas

**DOI:** 10.11604/pamj.2017.28.48.12049

**Published:** 2017-09-20

**Authors:** Fatima Ezzaitouni, Youssef Thiyfa, Mohamed Tahiri, Fouad Haddad, Wafaa Hliwa, Ahmed Bellabah, Wafaa Badre

**Affiliations:** 1 Service d’Hépato-Gastro-Entérologie, CHU Ibn Rochd, Casablanca, Maroc

**Keywords:** Déficit immunitaire commun variable, manifestations gastro-intestinales, infection, vitiligo, Common variable immunodeficiency, gastrointestinal manifestation, infection, vitiligo

## Abstract

Le déficit immunitaire commun variable (DICV) est une pathologie rare, il s’agit d’un déficit constitutionnel de l'immunité humorale caractérisé par des infections bactériennes à répétition, et par une fréquence accrue de tumeurs, de maladies auto-immunes ou granulomateuses. Les manifestations gastro-intestinales sont très variables et parfois révélatrices d’un déficit immunitaire commun variable. Nous rapportons le cas d’un patient âgé de 31 ans, ayant comme antécédent des infections respiratoires à répétition depuis le bas âge, compliquées d’une dilatation des bronches, qui présente depuis 3 ans une diarrhée glaireuse récurrente, chez qui l’ensemble du bilan étiologique est en faveur d’un DICV avec une manifestation auto-immune (vitiligo). Le traitement a consisté en une perfusion mensuelle d’immunoglobulines (Ig) avec une bonne évolution. Le recul actuel est de deux ans.

## Abstract (en)

Common Variable Immune Deficiency (CVID) is rare. It is a constitutional deficit of humoral immunity characterized by recurrent bacterial infections and by increased frequency of tumors, autoimmune or granulomatous diseases. Gastrointestinal manifestations are very variable and sometimes reveal common variable immune deficiency. We report the case of a 31-year old patient with a history of childhood recurrent respiratory infections complicated by bronchiectasis and with a 3-year history of recurrent glairy diarrhea. Etiological balance was in favor of CVID with autoimmune manifestation (vitiligo). Patient’s treatment was based on monthly immunoglobulin (Ig) infusions with favorable outcome at 2-year follow-up.

## Introduction

Le DICV est caractérisée par un défaut de production d’anticorps responsable d’une hypogammaglobulinemie d’expression variable. Il est définit par une diminution d’au moins deux écarts-types des taux sériques d’au moins deux classes d’Ig, en absence d’autres causes secondaires d’hypogammaglobulinemie [1]. Le DICV touche les hommes et les femmes de manière équivalente, avec une origine génétique variable, dont la physiopathologie n’est pas bien élucidée [2]. Le nombre des lymphocytes B (Lym B) circulants peut être normal, voire augmenté, et leur phénotype correspond à celui de cellules B matures (CD19+, CD20+, CD10+). Récemment, l’existence d’anomalies du mécanisme d’hypermutation somatique des gènes des Ig a été démontrée chez certains patients. Les manifestations gastro-intestinales sont très variables dont l’incidence varie de 20 à 60%, parfois révélatrices d’un déficit immunitaire commun variable avec certaines particularités histologiques et thérapeutiques [1].

## Patient et observation

**Figure 1:**
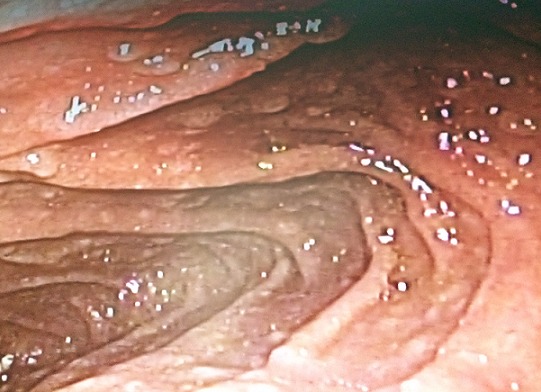
Aspect endoscopique micronodulaire de la muqueuse bulbo-duodénale

**Figure 2:**
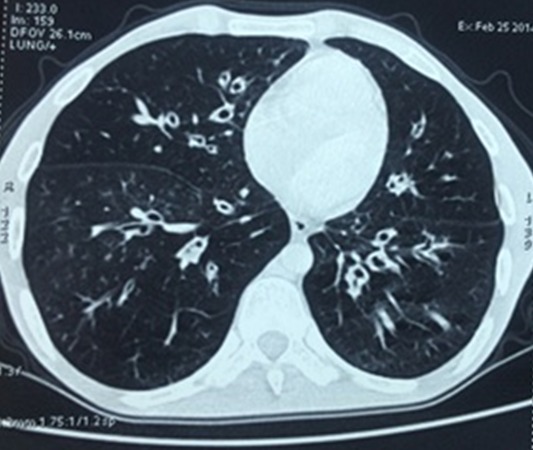
Fenêtre parenchymateuse du scanner thoracique montrant une dilatation des bronches cylindrique et bilatérale

Un jeune marocain âgé de 31 ans, ayant comme antécédent des infections respiratoires à répétition depuis le bas âge, qui présente depuis 3ans une diarrhée glaireuse récurrente à raison de 8-10 selles/j, évoluant dans un tableau d’altération de l’état général (score OMS à 4). L’examen clinique trouve un patient dénutri, avec des adénopathies cervicales, des râles ronflants bilatéraux et des tâches achromiques au niveau du visage et au tronc en rapport avec un vitiligo. Le bilan biologique montre un syndrome de malabsorption avec une hypocalcémie à 2,1 mmol/L, une hypocholestérolémie à 1,01 mmol/L, une hypokaliémie à 2,6 mmol/L et un syndrome inflammatoire avec une CRP à 57mg/l et une hyperfibrinogénémie à 9g/l. La numération formule sanguine objective une hyperleucocytose à 13400/mm^3^ à prédominance PNN (70%). La recherche de bactéries et de parasites dans les selles est négative. La sérologie du virus de l'immunodéficience humaine (HIV) est négative. La colonoscopie avec des biopsies coliques étagées a montré une muqueuse colique d’aspect endoscopique normal, avec à l’histologie une inflammation subaigüe et chronique évolutive non spécifique. La fibroscopie haute a mis en évidence une gastrite atrophique avec un aspect micronodulaire bulbo-duodénal (Figure 1), les biopsies gastriques ont conclu à une atrophie gastrique avec une métaplasie intestinale. Le nombre de lymphocytes intra-épithéliaux était inférieur à 30 lymphocytes pour 100 cellules épithéliales avec une positivité faible de phénotype CD4. La recherche des anticorps anti-endomysium et anti-transglutaminase type IgG est négative. L´électrophorèse des protides sériques montrait une diminution des gammaglobulines sanguines à 2,14 g/L, avec un déficit global en immunoglobulines (IgA à 0,0g/L (0,90-4,50), IgG à 1,29g/L (6,6– 12,8), et un taux normal des IgM à 0,93 g/L (0,50- 2,10). L’étude des sous-populations lymphocytaires ne montre pas d´anomalie qualitative ou quantitative. Le scanner thoracique montre une dilatation des bronches bilatérale (Figure 2). L´ensemble de ces éléments est en faveur d’un DICV avec une manifestation auto-immune type vitiligo. Le patient est mis sous substitution en immunoglobulines à 0,8g/kg en perfusion lente toutes les 4 semaines, une antibioprophylaxie par trimithoprime-sulfamétoxazole et une kinésithérapie respiratoire. L´évolution est favorable, avec une prise de poids, la disparition de la symptomatologie digestive et le syndrome bronchique. Le bilan de contrôle lipidique, hydro-électrolytique et radiologique est normal. Le recul actuel est de deux ans.

## Discussion

Le DICV est une pathologie rare de révélation tardive touchant entre 1/50 000 à 1/100 000 à l’échelon mondial [1]. Le DICV est encore plus rare en Afrique, puisque, seulement trois études à faible effectif (une égyptienne, une tunisienne et une algérienne) ont été publiées. Le DICV touche les hommes et les femmes de manière équivalente. Le diagnostic peut être posé à n’importe quel âge, mais le pic de fréquence se situe entre 20 et 30 ans. Les premiers épisodes infectieux surviennent le plus souvent dans l’enfance ou l’adolescence [1]. Dans la série américaine de Cunningham-Rundles et al [2] portant sur 248 patients, l’âge de début des symptômes suit une courbe bimodale, avec deux pics situés dans la première et la troisième décennie. Ces constatations ne sont pas retrouvées dans la série européenne (413 patients) [3] où les premiers symptômes pouvaient apparaître à tout âge avec une moyenne de 35 ans. L’âge de diagnostic chez notre patient est de 30 ans alors que les épisodes infectieux ont commencés depuis le bas âge.

La physiopathologie est très partiellement connue. Les lymphocytes B semblent immatures sans qu’on puisse clairement identifier d’anomalies intrinsèques; le défaut de production des Ig serait lié à des anomalies fonctionnelles de la coopération entre les lymphocytes T (Lym T) et B. Ces anomalies sont probablement hétérogènes, d’origine génétique différente d’un malade à l’autre expliquant des formes cliniques différentes [4]. Les bases génétiques du DICV restent inconnues bien que, de façon similaire au déficit sélectif en IgA, des loci de susceptibilité ont été retrouvés au niveau du CMH III sur le chromosome 6. Des mutations des gènes ICOS (inducible costimulator), codant pour une molécule d’interaction des lymphocytes T et B, et TACI (transmembrane activator and calcium modulator and cyclophilin ligand interactor) ont également été retrouvées chez les DICV à transmission autosomique récessive observée dans 10% des cas [1]. Les présentations cliniques sont multiples. Les infections représentent la principale manifestation des DICV, avec essentiellement des infections pulmonaires conduisant à de fréquentes bronchectasies. Les atteintes digestives sont à type de malabsorption, de maladie de « crohn-like », d’hyperplasie lymphoïde nodulaire et de giardiases. Des tableaux de granulomatose multisystémique, parfois pseudo-sarcoïdosiques, sont décrits. L’augmentation du risque relatif de cancers, principalement lymphomes et cancers du tractus digestif, est confirmée dans une étude récente [5].

Paradoxalement, dans cette affection caractérisée par un défaut de production d’anticorps, des manifestations auto-immunes (MAI) sont fréquemment associées, atteignant environ 25% des patients, principalement les femmes [2]. Les manifestations gastro-intestinales sont très variables, fréquentes (20–60%), et elles peuvent être souvent révélatrices du DICV [6, 7]. Il peut s’agir d’infections intestinales, de pathologies gastriques, grêliques ou recto-coliques. Mais ils sont principalement dominés par une diarrhée chronique ou récurrente. Cette diarrhée est souvent multifactorielle, liée à des infections chroniques surtout à Giardia lamblia mais peuvent également être dues à des atteintes spécifiques de la muqueuse intestinale comme les atrophies villositaires mimant la maladie cœliaque. De plus, les patients atteints de DICV sont à haut risque de cancers et de lymphomes digestifs [7]. Dans la plus large étude prospective et multicentrique d’Oksenhendler et al [8], incluant 252 patients, les infections intestinales sont les plus fréquentes. Les germes responsables sont surtout la Giardia, la Salmonella, le Campylobacter, le C. difficile et la yersinia. Leur diagnostic repose sur la coproculture et l’examen parasitologique des selles. Cependant, la sensibilité de l’examen parasitologique des selles pour l’isolement du trophozoïte ou des kystes de G.lamblia n’est que de 50%. La sensibilité de détection a été considérablement augmentée par les techniques d’Elisa (environ 90%) et plus encore par réaction en chaîne par polymérase (PCR), même si ces méthodes restent peu utilisées en pratique courante [1].

Il a été rapporté que la prévalence des gastrites à H.Pylori en cas de DICV est plus faible que celle habituellement observée dans la population générale occidentale (45 versus 87%) [9]. L’utilisation fréquente d’antibiotiques pour traiter les infections ORL et broncho-pulmonaires à répétition chez ces patients pourrait expliquer cette faible prévalence de gastrites à H. Pylori. Les atrophies villositaires sont très fréquentes, observées chez 31 à 60% des DICV présentant une anémie ou des symptômes gastro-intestinaux [9]. Cependant, la pathogénie de cette atrophie villositaire semble différente selon le type de déficit immunitaire. Les atrophies villositaires retrouvées au cours des déficits en IgA correspondent à de véritables maladies cœliaques. Par contre, l’association maladie cœliaque et DICV reste plus discutée. Si l’atrophie villositaire de DICV s’accompagne, comme au cours de la maladie cœliaque, d’une augmentation significative des lymphocytes intra-épithéliaux, elle se caractérise également par une raréfaction plasmocytaire du chorion [1]. De plus, le régime sans gluten, habituellement efficace dans le traitement des atrophies villositaires des déficits en IgA, s’avère peu efficace sur les atrophies villositaires des DICV [9].

Une hyperplasie folliculaire lymphoïde (HFL) est observée chez près de 60% des DICV. À l’inverse des HFL banales du sujet jeune immunocompétent, localisées à l’iléon terminal et au colon droit, les HFL des DICV sont volontiers diffuses à tout l’intestin grêle et au colon. Dans ce contexte, les HFL pourraient favoriser la survenue de proliférations lymphomateuses chez ces patients, la présence d’HFL « atypique », nid du lymphome intestinal, est en effet rapporté. Les lymphomes digestifs semblent fréquents au cours du DICV avec les autres cancers digestifs, soit 50% des DICV ayant une pathologie maligne avec un risque plus élevé par rapport à la population générale. La majorité des lymphomes intestinaux survenant chez les DICV sont des lymphomes non hodgkiniens de phénotype B. Le risque de cancer gastrique serait 50 fois supérieur à celui de la population générale, les gastrites atrophiques ont été décrites comme le principal facteur favorisant, ce risque a pu être surévalué puisque des études plus récentes rapportent un risque à 10 fois celui de la population générale [10]. Cependant la prévalence de l’adénocarcinome colique ne semble pas augmenter au cours du DICV [1]. Une colite chronique est décrite au cours du DICV, résultante d’une hyperlymphocytose intra-épithéliale qui s’associe à un infiltrat inflammatoire de la lamina propria et de l’épithélium glandulaire et s’accompagne d’une raréfaction cryptique, mimant ainsi l’aspect histologique observé au cours des maladies inflammatoires chroniques de l’intestin, dont la fréquence semble augmentée [1, 11]. Mais contrairement aux maladies inflammatoires chroniques de l’intestin de l’immunocompétent, celles des DICV sont caractérisées par la raréfaction, voire l’absence, des plasmocytes du chorion [11], chez le cas rapporté dans notre observation, on note une raréfaction du plasmocyte CD4 au cours de l’étude immunohistochimique des biopsies duodénales. Une pneumonie bactérienne est observée chez 63 à 86% des malades atteints de DICV [4, 7] et devance très largement en fréquence les complications infectieuses non pulmonaires. Elle est souvent inaugurale et récidivante. Avant le traitement substitutif, elle était responsable de plus de 1 épisode chez 68% et de plus de 10 épisodes chez 18% des malades de la série de Hermans et coll [7]. Elle est pratiquement toujours en rapport avec des bactéries encapsulées. Streptococcus pneumoniae et Haemophillus influenzae représentaient respectivement 37 % et 26 % des germes identifiés dans une série récente [4].

Une suppuration bronchique clinique est notée chez 17 à 28% des malades [4, 7]. Elle est indissociable de la suppuration des voies aériennes supérieures (otite moyenne et sinusite) qui est observée chez 36 à près de 100 % des malades. Des anomalies évocatrices de dilatation des bronches (DDB) sur la radiographie de thorax sont également présentes dans 14 à 38% des cas [12] au moment du diagnostic et sont observées en tomodensitométrie chez 42 à 79 % des malades. La DDB est le plus souvent de type cylindrique (88 à 100%), diffuse et bilatérale (60 à 75 %), mais sans prédominance basale ou apicale, comme c’est le cas chez notre patient. Il existe très fréquemment des signes d’atteinte bronchiolaire et des atélectasies en rapport avec des impactions mucoïdes. Hemophilus influenzae est le germe le plus souvent responsable d’exacerbations bronchiques aiguës [4] ; il est pratiquement toujours résistant à l’amoxicilline du fait de la sécrétion d’une bêtalactamase. Des colonisations à Staphylococcus aureus ou Pseudomonas aeruginosa apparaissent plus tardivement dans l’évolution de la maladie [12]. A cote des classiques manifestations infectieuses et digestives, les MAI émaillent l’évolution de 24 à 48% des patients ayant un DICV [13]. Dans une série algérienne, les chiffres obtenus (17 %) semblent sous-estimer la fréquence réelle de ces manifestations du fait de l’absence d’une recherche systématique des MAI chez les patients. La cytopénie auto-immune, est la plus fréquemment retrouvée au cours du DICV [13], dans la série de Cunningham-Rundles [2], 40% des 56 patients atteints de DICV et MAI ont une cytopénie auto-immune, soit 9% des 248 patients étudiés. Dans une étude multicentrique française, portant sur 9 patients atteints de DICV et de MAI [5], 77% ont une cytopénie auto-immune dont trois anémies hémolytiques auto-immunes, deux purpuras thrombopéniques idiopathiques (PTI) et deux syndromes d’Evans. D’une façon moins prédominante, d’autres MAI telles que le vitiligo, le psoriasis, la thyroïdite de Hashimoto, l’anémie pernicieuse, la maladie cœliaque, la polyarthrite rhumatoïde, l’arthrite juvénile idiopathique, le lupus érythémateux systémique, le syndrome de Sjogren peuvent aussi être retrouvées au cours du DICV [5, 13]. La survenue de la MAI peut précéder de plusieurs mois, voire plusieurs années, le diagnostic du DICV. De la même manière, le DICV n’est pas connu au moment du diagnostic de PTI dans 16 des 18 cas rapportés par Cunningham-Rundles [2]. Ainsi, il serait intéressant de rechercher une hypogammaglobulinemie devant une cytopénie auto-immune ou une autre MAI. Dans la série du Pavic et al [5], l’âge moyen au diagnostic de la MAI est de 27 ans. Le diagnostic de MAI est antérieur, de un à 18 ans, à celui de DICV dans cinq cas. L’association cirrhose biliaire primitive et myasthénie est observée chez une patiente ayant des antécédents familiaux de deux fils porteurs d’un DICV et d’un frère porteur d’un diabète de type 1 [5]. La fréquence de la maladie granulomateuse au cours du DICV varie de 8 à 22% selon les séries [2, 14]. Les localisations peuvent être multiples (ganglions, rate, foie, peau, tube digestif, parenchyme pulmonaire, moelle osseuse mais aussi cérébrale). La maladie granulomateuse pourrait en imposer pour une sarcoïdose (certains auteurs préfèrent parler de réactions «sarcoïdose-like») mais l’hypogammaglobulinemie doit faire suspecter un DICV, ce d’autant qu’au cours des sarcoïdoses « classiques » les gammaglobulines sont le plus souvent élevées [4, 13].

Le diagnostic de DICV ne peut être porté qu’après exclusion des autres causes d’hypogammaglobulinèmie tels que l’entéropathie exsudative, le syndrome néphrotique, un état de dénutrition grave…Le bilan nécessaire pour poser le diagnostic d’un DICV comporte une électrophorèse des protides, un dosage pondéral des Ig, une numération des lymphocytes B circulants et un phénotypage lymphocytaire (Lym T( CD3, CD4, CD8) et Lym B (CD19 ,CD20, CD27)). En cas de normalité du taux des Ig et/ou en cas d’infections à répétition, un dosage des sous-classes d’IgG est recommandé, car le déficit peut ne concerner que les IgG2, dont la baisse pourrait passer inaperçue lors d’un dosage global des IgG [1]. Le retentissement de la maladie est évalué par la recherche de foyers infectieux chroniques, surtout dentaires, oto-rhino-laryngés, bronchopulmonaires et digestifs, mais aussi urinaires, cutanés, articulaires, neurologiques ou septicémiques. La répétition des épisodes infectieux pulmonaires peut évoluer vers la dilatation des bronches et l’insuffisance respiratoire chronique [1, 4].

L’hétérogénéité des présentations cliniques et immunophenotypiques du DICV a conduit à établir des classifications afin de définir des groupes homogènes de patients [13]. Bryant et al, ont proposé une classification fonctionnelle établie à partir de la capacité de production des IgM, IgA et IgG par les lymphocytes B en culture in vitro en réponse à un agent stimulant. Cette classification est controversée en raison de problèmes techniques et du manque de corrélation avec les phénotypes lymphocytaires et les caractéristiques cliniques [15]. Warnatz et al. proposent une autre classification fondée, cette fois-ci, sur l’immunophenotypage par cytométrie en flux des Lym B CD19+ circulants, mesurant l’expression du CD27, marqueur des lymphocytes B mémoires, et du CD21, dont le niveau d’expression reflète le stade de maturation des cellules B naïves nouvellement formées sortant de la moelle osseuse. Deux groupes de patients sont alors individualisés en fonction du nombre de Lym B mémoires: groupe I ayant moins de 0,4% de Lym B mémoires (par rapport aux Lym totaux) et groupe II ayant plus de 0,4%. Le groupe I est, lui-même, divise en deux sous-groupes: le sous-groupe Ia ayant plus de 20% de Lym B CD21 et le sous-groupe Ib ayant moins de 20% de Lym B CD21. Les patients du groupe I présentent généralement une maladie plus sévère; en particulier ceux du sous-groupe Ia ont une incidence accrue de la splénomégalie [16].

**Figure 3:**
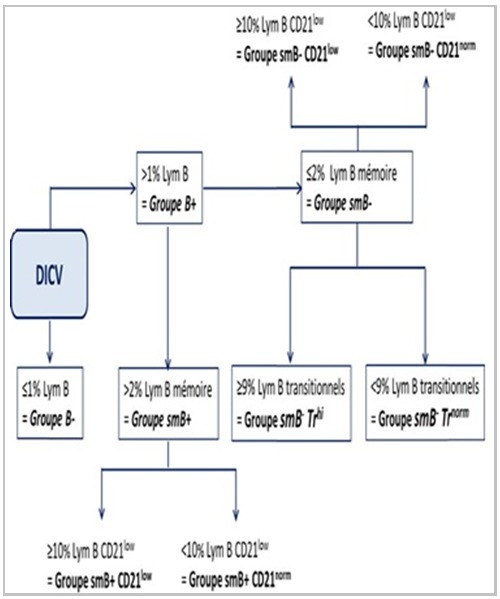
CLassification consensus du DICV (EUROClass classification)

En 2007, une autre classification « The EUROClass classification » est proposée. Cette classification consensus actuellement distingue deux principaux groupes: le groupe B- caractérisé par un taux de Lym B < 1% et le groupe B+ ayant un taux de lymphocytes B > 1%, avec des sous-groupes (Figure 3). Le groupe B+ est ensuite subdivisé en deux: groupe smB- avec un taux de Lym B mémoires commutés (CD19+ CD27+ IgM- IgD-) moins de 2% (par rapport aux Lym B CD19+) et le groupe smB+ ayant un taux de Lym B mé moires commutés > 2%. Les patients du groupe smB- sont ensuite subdivisés en deux sous-groupes: smB-Trhi avec plus de 9% de Lym B transitionnels (CD21int CD38++ IgM++) et smB-Trnorm (< 9% de Lym B transitionnels). Cette classification distingue aussi deux groupes supplémentaires selon la présence ou non d’une expansion des Lym B CD21low: groupe CD21low (Lym B CD21low Ç 10%) et groupe CD21norm (Lym B CD21low < 10%). Sur le plan clinique, l’appartenance au groupe smB- expose à un risque plus élevé d’apparition de splénomégalie et de maladie granulomateuse [17].

Le traitement du DICV est un traitement substitutif en immunoglobulines qui prévient les infections bactériennes et virales. Ce traitement est, en règle, administré à vie. Il repose sur des perfusions d’Ig intraveineuse et plus récemment sur des perfusions à administration sous-cutanée, permettant ainsi d’améliorer la qualité de vie avec une pharmacocinétique similaire à la voie intraveineuse [1, 18]. Il n’existe pas de protocole consensuel, mais il est classiquement recommandé de commencer par un bolus d’Ig intraveineuse à la dose de 1 mg/kg, suivi soit par des perfusions intraveineuses mensuelles à la dose de 0,4 g/kg, soit par des perfusions sous-cutanées hebdomadaires à la dose de 0,1 à 0,2 g/kg [18]. L’objectif est d’atteindre un taux résiduel de gammaglobulines sériques de 5 g/l. Les perfusions sont en règle bien tolérées, et chez les patients ayant un déficit associé en IgA, il faut rechercher des anticorps anti-IgA d’isotype IgG ou IgE, qui, s’ils sont présents, peuvent être à l’origine de réactions anaphylactiques [1]. Le traitement des manifestations digestives est spécifique à chaque situation. Le traitement des infections à Giardia lamblia repose sur l’utilisation répétée de métronidazole ou de tinidazole, car ces infections sont volontiers récidivantes et résistantes au traitement antibiotique. L’effet de la substitution intraveineuse en Ig seule, semble peu efficace sur la diarrhée et les infections intestinales [1, 9, 19].

En présence d’atrophie villosiatire, un régime sans gluten pourrait être indiqué, bien que l’association à une maladie cœliaque semble exceptionnelle. En cas de résistance au régime, le diagnostic d’atrophie villositaire primitive du DICV est retenu, nécessitant parfois le recours à une alimentation parentérale, à une corticothérapie, voire à l’utilisation de ciclosporine [20]. Dans certains cas, le budésonide pourrait être efficace sur les atrophies villositaires des DICV résistantes au régime sans gluten [9]. En cas de manifestations respiratoires, une antibioprophylaxie à base de trimethoprime sulfamétoxazole peut être proposée, et une kinésithérapie respiratoire si dilatation de bronches associée.

## Conclusion

Le DICV constitue un groupe hétérogène de pathologies rares et complexes, comportant un risque accru de cancers digestifs et de lymphomes. Les manifestations gastro-intestinales sont fréquentes et souvent révélatrices du DICV, en particulier chez l’adulte jeune. La constatation d’une atrophie villositaire séronégative ou résistante au régime sans gluten, ou de maladies inflammatoires chroniques de l’intestin résistantes aux traitements habituels doit faire rechercher un DICV. Une prise en charge précoce permet une réduction de la morbidité et de la mortalité.

## Conflits d’intérêts

Les auteurs ne déclarent aucun conflit d'intérêt.
